# Inhibition of Toxic Shock by Human Monoclonal Antibodies against Staphylococcal Enterotoxin B

**DOI:** 10.1371/journal.pone.0013253

**Published:** 2010-10-11

**Authors:** Eileen A. Larkin, Bradley G. Stiles, Robert G. Ulrich

**Affiliations:** 1 Immunology Department, Medical Research Institute of Infectious Diseases, Frederick, Maryland, United States of America; 2 Biomedical Sciences Department, Hood College, Frederick, Maryland, United States of America; 3 Biology Department, Wilson College, Chambersburg, Pennsylvania, United States of America; University of Liverpool, United Kingdom

## Abstract

**Background:**

*Staphylococcus aureus* is implicated in many opportunistic bacterial infections around the world. Rising antibiotic resistance and few alternative methods of treatment are just two looming problems associated with clinical management of *S. aureus*. Among numerous virulence factors produced by *S. aureus*, staphylococcal enterotoxin (SE) B is a secreted protein that binds T-cell receptor and major histocompatibility complex class II, potentially causing toxic shock mediated by pathological activation of T cells. Recombinant monoclonal antibodies that target SEB and block receptor interactions can be of therapeutic value.

**Methodology/Principal Findings:**

The inhibitory and biophysical properties of ten human monoclonal antibodies, isolated from a recombinant library by panning against SEB vaccine (STEBVax), were examined as bivalent Fabs and native full-length IgG (Mab). The best performing Fabs had binding affinities equal to polyclonal IgG, low nanomolar IC_50_s against SEB in cell culture assays, and protected mice from SEB-induced toxic shock. The orthologous staphylococcal proteins, SEC1 and SEC2, as well as streptococcal pyrogenic exotoxin C were recognized by several Fabs. Four Fabs against SEB, with the lowest IC_50_s, were converted into native full-length Mabs. Although SEB-binding kinetics were identical between each Fab and respective Mab, a 250-fold greater inhibition of SEB-induced T-cell activation was observed with two Mabs.

**Conclusions/Significance:**

Results suggest that these human monoclonal antibodies possess high affinity, target specificity, and toxin neutralization qualities essential for any therapeutic agent.

## Introduction

*Staphylococcus aureus* is a common source of many diseases for both humans and domestic animals [1]. This bacterium presents a daunting medical problem due to increasing antibiotic resistance among nosocomial- and community-acquired isolates [2]–[4]. The economic burden of *S. aureus* upon healthcare systems around the world is substantial and new means of controlling diseases caused by this pathogen are clearly needed now [5], [6].

To gain an infective foothold, *S. aureus* produces several factors that facilitate adherence, interfere with a proper immune response, or otherwise alter the host microenvironment. One type of virulence factor includes the staphylococcal enterotoxins (SEs), originally distinguished by serotyping methodology [7]–[10]. These protein toxins can cause acute gastroenteritis and toxic shock syndrome. Although SEs (>20 known to date) appear distinct by amino acid sequence comparisons [11], all share common superantigen structures consisting of an N-terminal OB (oligonucleotide/oligosaccharide binding) fold and C-terminal, ubiquitin-like, beta-grasp domain. Related superantigenic proteins are also expressed by another bacterial pathogen, *Streptococcus pyogenes* [12]. SE cross-linking of major histocompatibility complex class II molecules (MHC II) and specific subsets of T-cell antigen receptors (TCR) activate the immune system [13]. Superantigenic effects [14] involve profound T-cell proliferation and elevated levels of the proinflammatory cytokines interferon gamma (IFNγ), interleukin 2 (IL-2), and tumor necrosis factor alpha (TNFα). Toxic shock syndrome, due to bacterial superantigen exposure, can rapidly progress to severe and intractable hypotension, multi-system failure and death.

Concerning toxin-induced diseases associated with *S. aureus*, the most comprehensive clinical data are available for staphylococcal toxic shock caused by toxic shock syndrome toxin-1 (TSST-1). There is a clear predisposition towards initial and recurring bouts of toxic shock syndrome among menstruating women lacking pre-existing antibodies against TSST-1 [15], [16]. Significant levels of TSST-1 antibodies are found amongst approximately 85% of menstruating women between 13 and 40 years of age [16]. In particular, TSST-1 specific IgG1 and IgG4 are protective whereas IgM and IgA reportedly have no effect [15]. Non-menstrual toxic shock, which can be more deadly than menstrual forms [17], has been linked to SEB, TSST-1, and other bacterial superantigens produced by *S. aureus* [18]. Experimentally, antibodies against superantigens can be neutralizing and induced by recombinant vaccines [19]–[23]. Furthermore, specific antibodies given passively to naïve mice or non-human primates concomitant to, or hours after, toxin exposure protect against toxic shock [24]–[26].

While concentrated preparations of non-specific human immunoglobulin (i.e. intravenous immunoglobulin or IVIg) are used to treat streptococcal- and staphylococcal-induced shock, clinical trials are rather limited to date [27], [28]. Preparations of these polyclonal antibodies from humans neutralize superantigens *in vitro* [29], [30], although other non-specific mechanisms may contribute to protection [31]. Reports also suggest that some IVIg preparations are simply not efficacious for streptococcal and staphylococcal infections [32]–[34]. Additionally, the IVIg used clinically may be more effective towards streptococcal than staphylococcal superantigens, and there is also a natural batch-to-batch variation [35], [36]. A step beyond IVIg involves clinical trials targeting a few select antigens from *S. aureus* through: 1) immunization of humans and subsequent collection of immunoglobulins; or 2) recombinant generation of antibodies. However, these more-targeted approaches are either currently being tested or have failed to perform [28].

Clearly, there is a need for well-characterized, safe and efficacious immunotherapeutics for treating diseases linked to staphylococcal superantigens like SEB. Recombinant human monoclonal antibodies can be manufactured under controlled conditions and are potential alternatives to IVIg. It is possible to select or engineer native-like monoclonal antibodies with almost any specificity by harnessing recombinant DNA technologies [37]. Furthermore, recipients are less prone to a life-threatening anaphylactic reaction [38] or anti-therapeutic antibody response, possible consequences of giving any foreign immunoglobulin to humans.

We selected human monoclonal antibodies from a phage-display library, using a recombinant SEB vaccine (STEBVax) incorporating site-specific mutations that prevent MHC II interactions [21]. Previous studies show that antibody responses to STEBVax protect rhesus macaques against toxic shock caused by wild-type SEB [21], [22]. In this current study, we discovered that some antibody clones cross-react with SEC1, SEC2, and streptococcal pyrogenic exotoxin C (SpeC), while others were highly specific for SEB. Many of the antibodies effectively inhibited T-cell activation by SEB *in vitro*, bound to toxin with nanomolar affinity, and prevented SEB-induced toxic shock *in vivo*.

## Results

### Antibody Specificity

Previous studies showed that rhesus macaques vaccinated with STEBVax, an SEB molecule containing MHC II-binding site mutations (L45R, Y89A, and Y94A), produced antibodies that prevented SEB-induced illness [20]–[22]. Reasoning that neutralizing antibodies could be obtained without requiring biocontainment facilities, we used STEBVax to pan the synthetic phage-display library and select recombinant human antibodies [39]. The probe antigen was biotinylated and bound to paramagnetic beads covalently modified with streptavidin to enable selection of antibodies in solution. Ten human Fabs thus selected were initially tested for reactivity with other bacterial superantigens by ELISA (Table 1). All STEBVax-panned Fabs, except #4, recognized wild-type SEB. Seven of the antibodies also recognized closely-related SEC1 and/or SEC2 [40], and five cross-reacted with SpeC, a pattern also exhibited by SEB affinity-purified IgG from human sera (Table 1). None of the antibodies reacted with SEA, TSST-1, or SpeA as measured by ELISA.

**Table 1:** Specificity of Human Monoclonal Fabs for Staphylococcal and Streptococcal Superantigens.

Antibody^a^	Antigen						
	SEA	SEB	SEC1	SEC2	TSST-1	SpeA	SpeC
1	-b	+++	+++	-	-	-	-
2	-	++++	-	-	-	-	-
3	-	++++	-	-	-	-	+
4	-	-	-	+	-	-	+
5	-	+++	-	-	-	-	-
6	-	++++	++++	+	-	-	++
7	-	+++	+++	-	-	-	-
8	-	+++	+++	-	-	-	-
9	-	++	-	+	-	-	++
10	-	++	+	++	-	-	++
Polyclonal IgG	-	+++	++	+++	-	-	++

a Numbered antibodies are monoclonal Fabs. Polyclonal IgG is anti-SEB affinity purified from human sera.

b Scoring by ELISA:

- = <0.5 mean absorbance +/− standard deviation.

+ = 0.5–1.0.

++ = 1.0–2.0.

+++ = 2.0–3.0

++++ = 3.0–4.0.

**Figure 1:**
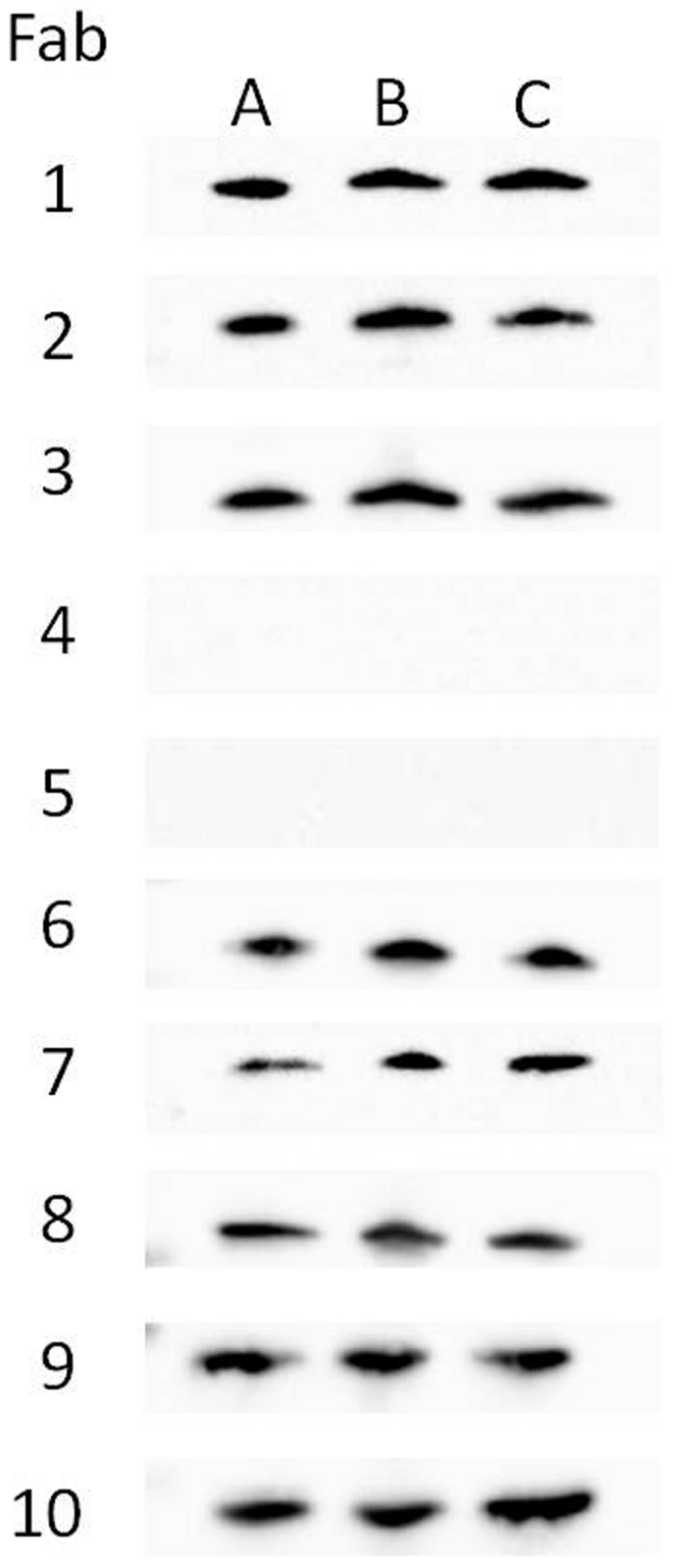
Antibody detection of SEB in a complex antigen mixture by Western blot. A 10–20% gradient SDS-PAGE separated crude *S. aureus* antigen preparations subsequently transferred onto nitrocellulose. Each Fab (1 µg/ml) was used to probe the nitrocellulose for 1 h. Lanes include: A- 50 ng purified SEB; B – 20 µg cell lysate; and C – 20 µg culture supernatant.

In addition to staphylococcal and streptococcal toxin recognition of Fabs by ELISA, Western blots were done with *S. aureus* culture fluid and cell lysate (Fig. 1). Eight of the ten Fabs specifically recognized SEB in complex antigen preparations from a previously characterized strain of *S. aureus*. Two Fabs did not detect SEB under these conditions, including Fab 4 that yielded similar results by ELISA. These two antibodies likely recognize conformation-dependent epitopes. In addition to the 1 h incubation of Fab with antigen (Fig. 1), there were similar, specific-banding patterns after only a 15 min incubation of primary antibody (data not shown).

### Kinetic Analysis of Antibody Binding to SEB

Interactions between immobilized antibody and solution-phase SEB were analyzed by SPR. Although all Fabs were tested, we were able to obtain complete data for antibodies 1, 3, 6, 9, and 10 (Table 2). The SPR results confirmed specific binding for antibodies 2, 4, 5, 7, and 8, but systematic deviation between the experimental and fitted curves, based on 1∶1 Langmuir binding, prevented accurate calculations of association and dissociation rates. Both Fab 10 and the affinity-purified polyclonal antibody exhibited similar nanomolar KDs and binding kinetics with no apparent correlation between antigen cross-reactivity (Table 1) and SEB-binding affinity (Table 2). Select Fabs were converted to full-length, native Mabs and the binding kinetics with SEB examined again. Antibody interactions with SEB appeared similar between each Fab and its native Mab derivative, suggesting minimal disturbance of epitope recognition by Fc addition (Table 2).

**Table 2:** Kinetic Analysis of Immobilized Human Antibody Interactions with SEB in Solution.

	Fab			Native Full-Length Mab		
Antibody^a^	ka (1/Ms)	kd (1/s)	KD (M)	ka (1/Ms)	kd (1/s)	KD (M)
1	4.7×10^3^ (+/−2.9×10^2^)^b^	6.9×10^−3^ (+/−2.3×10^−4^)	1.7×10^−6^ (+/−1.7×10^−7^)	6.2×10^3^ (+/−8.3×10^2^)	6.7×10^−3^ (+/−5.2×10^−4^)	1.1×10^−6^ (+/−2.3×10^−7^)
3	1.5×10^4^ (+/−1.8×10^2^)	3.3×10^−4^ (+/−3.3×10^−6^)	2.3×10^−8^ (+/− 0)	5.0×10^4^ (+/−2.6×10^3^)	3.7×10^−4^ (+/−5.7×10^−5^)	7.5×10^−9^ (+/−7.4×10^−10^)
6	96.9 (+/−2.5)	4.1×10^−5^ (+/−4.2×10^−6^)	4.2×10^−7^ (+/−4.4×10^−8^)	nt^c^	nt	nt
9	7.3×10^3^ (+/−9.4×10^2^)	1.6×10^−4^ (+/−2.0×10^−5^)	2.2×10^−8^ (+/−5.0×10^−11^)	1.7×10^4^ (+/−2.8×10^3^)	1.8×10^−4^ (+/−2.2×10^−5^)	1.1×10^−8^ (+/−1.1×10^−9^)
10	2.7×10^4^ (+/−2.1×10^3^)	1.2×10^−4^ (+/−1.6×10^−5^)	4.2×10^−9^ (+/−2.8×10^−10^)	5.1×10^4^ (+/−1.3×10^4^)	6.3×10^−5^ (+/−3.6×10^−6^)	1.3×10^−9^ (+/−2.5×10^−10^)
Polyclonal IgG	nt	nt	nt	3.2×10^5^ (+/−4.9×10^3^)	3.3×10^−4^ (+/−5.3×10^−5^)	1.0×10^−9^ (+/−1.8×10^−10^)

a Antibody immobilized on a CM5 chip with SEB used as analyte. Polyclonal IgG was SEB affinity-purified IgG from human sera.

b Standard deviation.

c Not tested.

### Antibody Inhibition of SEB Binding to MHC II

From a therapeutic perspective, it was important to ascertain if any of the recombinant antibodies interfered with SEB binding to receptor in a biological assay. Antibody-mediated inhibition of toxin binding to either MHC II or TCR can afford protection. We examined antibody inhibition of SEB - MHC II interactions using an LG2 cell-based assay and flow cytometry analysis (data not shown). Only Fabs 9 and 10 effectively prevented SEB binding (∼95%) to MHC II, and these antibodies were equivalent in this assay to the SEB-purified IgG from human sera used as a control. Fab 1 weakly (∼20%) inhibited SEB binding to MHC II while Fabs 2 - 8 had no effect. There was no inhibition of SEB binding to MHC II by a control Fab (SEA-selected clone). Furthermore, direct comparison of parental Fabs with native-length Mabs 9 and 10 revealed no differences in blocking SEB binding to MHC II by this assay.

### Antibody Inhibition of T-cell Response to SEB

We next addressed toxin-neutralizing capabilities of the antibodies in cell culture. Activation of T lymphocytes in human mononuclear cell cultures was monitored by measuring release of IFNγ in response to SEB pre-incubated with each antibody. By IC_50_ determination, the most potent (SEB-neutralizing) Fabs were 1, 3, 9 and 10, whereas Fabs 7 and 8 had no inhibitory effect (Table 3). Although the positive antibody control (SEB-specific IgG from human sera) effectively inhibited T-cell activation by SEB, Fab 10 was slightly better. A negative control Fab against SEA, which did not react with SEB by ELISA, had no effect upon SEB-induced activation of T cells.

**Table 3:** Antibody Inhibition of Human T-Cell Responses to Bacterial Superantigens.

	IC_50_ (nM).		
Antibody a	SEB	SEC1	SpeC
Fab 1	205	1487	nt^b^
FL 1	100	nt	nt
Fab 2	1255	nt	nt
Fab 3	199	nt	>1500 (3)^c^
FL 3	241	nt	nt
Fab 4	558	nt	>1500 (0)
Fab 5	772	nt	nt
Fab 6	875	480	nt
Fab 7	>1500 (6)	>1500 (23)	nt
Fab 8	>1500 (9)	>1500 (20)	nt
Fab 9	444	nt	769
FL 9	1.44	nt	916
Fab 10	77	nt	>1500 (39)
FL 10	0.32	nt	>1000 (0)
Anti-SEB^d^	92	570	59
SEA Fab^e^	>1500 (0)	nt	nt

a FL  =  full-length native Mab. All data show antibody inhibition of toxin-induced IFNγ release, relative to toxin only-treated PBMCs that act as a control. Data represent the mean of quadruplicate readings resulting from two separate experiments.

b Not tested, due to weak cross-reactivity by ELISA.

c Percent inhibition given in parentheses at the highest concentration (1500 nM for Fab and 1000 nM for FL Mab) of weak-performing antibodies.

d Polyclonal anti-SEB from human sera, affinity-purified against SEB.

e Control human Fab against SEA that did not cross-react with SEB by ELISA.

To further complement the SEB studies, we also examined Fab inhibition of T-cell responses to other cross-reacting superantigens. Fab 6 most effectively inhibited SEC1-induced responses, as monitored by IFNγ secretion, and was slightly more potent than the polyclonal IgG (Table 3). Uniquely, the IC_50_ of Fab 6 against SEC1 was approximately two-fold better than that for SEB. Among the remaining cross-reacting antibodies, Fab 1 had a barely-detectable effect against SEC1. Although six of the Fabs recognized SpeC by ELISA (Table 1), only Fab 9 had any significant effect on SpeC-induced stimulation of T-cells. In contrast to the recombinant antibodies, polyclonal IgG more effectively inhibited SpeC, versus SEB or SEC1, activation of human T cells (Table 3). Altogether, these results indicated that common, neutralizing epitopes on staphylococcal and streptococcal superantigens are recognized by this panel of human monoclonal antibodies.

The SPR-based results (Table 2) indicated that interactions with SEB were similar between Fab and native-structured Mab. Therefore, we next examined T-cell responses to see if the antibody form influenced inhibition of SEB. Our results indicated that the IC_50_s for native-length Mabs 1 and 3 were essentially the same as the parental Fabs by cellular assay (Table 3). In sharp contrast, the IC_50_s of Mabs 9 and 10 against SEB were significantly (∼250-fold) better than the parental Fab. However, this effect was not apparent with SpeC. Further comparisons revealed that Mabs 9 and 10 were respectively 65- and 280-fold more potent than polyclonal IgG against SEB.

**Figure 2:**
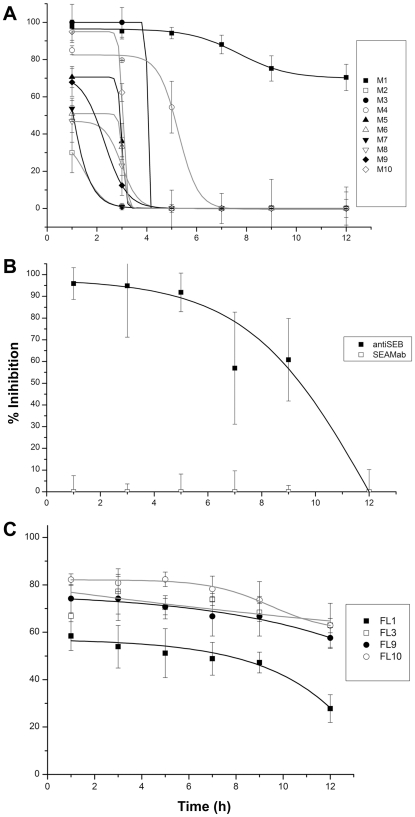
Therapeutic capabilities of antibodies added after SEB in human PBMC cultures. Fab or native full-length (FL) Mabs were used at a final concentration of 1000 nM and added at designated times after SEB (0.35 nM). Culture fluids were collected 16 h after SEB addition and IFNγ concentrations ascertained as described in the Materials and Methods. Panels A and C respectively show Fab and FL-Mab results. Antibody controls consisted of a SEA-specific Fab and SEB affinity-purified IgG from human sera (Panel B). Results are representative of two separate experiments.

Upon establishing that select Fabs prevented superantigen-induced stimulation of human T-cells when premixed with toxin, we next examined delayed addition of antibody. Human PBMCs were cultured with SEB (0.35 nM), and Fabs (1000 nM) were then added to wells at designated time intervals (Fig. 2A). Preliminary studies investigating IFNγ release, following toxin stimulation of PBMCs, revealed that IFNγ is undetected in culture fluid until 7 h after toxin addition. There is a subsequent linear increase in IFNγ concentrations that plateaus and remains stable between 22–48 h after toxin exposure (data not shown). Inhibitory profiles could be separated into Fabs that blocked IFNγ release ≤5 h after SEB addition, or Fabs inhibiting T-cell responses >5 h. Most noteworthy, SEB-induced T-cell responses were inhibited 75% by Fab 1 up to 12 h after SEB exposure (Fig. 2A), whereas inhibition by the SEB affinity-purified IgG dropped to 0% by 12 h (Fig. 2B). An SEA-specific, control Fab had no effect on T-cell responses (Fig. 2B). The inhibition range of SEB Fabs at 1 h varied from 30–100%, and by 3 h spanned 0–97% (Fig. 2A). Fab 4 was slightly better than all others except Fab 1, inhibiting 50% of the IFNγ release at 5 h post-SEB.

In contrast to the results obtained with Fabs (Fig. 2A), the native-length Mabs 3, 9, and 10 inhibited 60 – 80% of T-cell responses up to 12 h after SEB (Fig. 2C). In a manner similar to Fab 1, native Mab 1 was also inhibitory but to a lesser extent (60–30%). Overall, these observations suggested that biological activity of the Fabs was influenced by added Fc in this assay.

To examine the role of Fc receptors, mouse L-cells devoid of Fc receptor were used to replace the mixed mononuclear cells in the IFNγ assay. The L cells were transfected with human MHC II (HLA-DR1α/β) for presentation of SEB to human T-cells. Results with the L-cell transfectants indicated that inhibition of SEB-induced T-cell responses by native Mab returned to a level equivalent to Fab, suggesting an important contribution of Fc receptors towards toxin neutralization (data not shown).

### Antibody Neutralization of SEB in Mice

To further study protective capabilities of select antibodies, we used a mouse model of SEB-induced toxic shock (Table 4). The results were compared to controls receiving SEB only (0% survivors) or SEB plus anti-SEA Fab (17% survivors). Fab 10 and its full-length native construct both provided the highest protection (68%) from toxic shock within the groups examined, followed by Fab 1 (42%) and Fab 9 (34%). These results were consistent with the IC_50_s of Fab 10< Fab 1< Fab 9 (Table 3). In contrast, Fab 3 and Mab 9 were no different than controls given anti-SEA Fab. While a more comprehensive study will be required to differentiate the biological effects of each antibody *in vivo*, these data are in general agreement with the collective *in vitro* results.

**Table 4:** Antibody Neutralization of SEB *in vivo*.

Antibody^a^	% Survival^b^
Fab 1	42
Fab 3	17
Fab 9	34
FL 9	17
Fab 10	68
FL 10	68
SEA Fab^c^	17
None	0

a FL  =  full-length native Mab.

b n = 12 for Fab 1, SEA Fab, and None. n = 6 for all other groups. Before intraperitoneal injection of each mouse, antibody (10 µg) was premixed with SEB (2.5 µg) for 30 min at 22°C. Survival was recorded over a 72 h period.

c Control human Fab against SEA and not cross-reacting with SEB by ELISA.

## Discussion

Virulence factor-specific antibodies derived from vaccination or employed as therapeutics represent a potential defense against bacterial diseases. Our results describe for the first time recombinantly-derived, human monoclonal antibodies against SEB that possess high affinity, target specificity, and therapeutic potential for superantigen-induced toxic shock. These antibodies prevent intoxication by interfering with toxin binding to MHC II and/or TCR. In addition to potential applications for treating toxic shock syndrome, human monoclonal antibodies recognizing SEB or other bacterial superantigens may be useful if employed as an adjunct therapy with antibiotics in treating difficult *S. aureus* infections. For example, a combination of antibiotics and immunoglobulins was previously advocated for combating *S. pyogenes* infections [41], [42]. This paradigm may be effective against methicillin-, as well as vancomycin-, resistant strains of *S. aureus* [3], [4], [43], [44] since many isolates in the United States produce various superantigens that include SEB and SEC [27].

Other antibody-based therapeutic approaches have been suggested for treating *S. aureus* infections. For example, a phase II study employing a humanized IgG1 targeting *S. aureus* clumping factor A (ClfA; a fibrinogen-binding protein) resulted in good tolerance by patients and promising preliminary clinical findings [45]. An IVIg preparation used clinically in neonates contains antibodies against microbial surface components that recognize adhesive matrix molecules (MSCRAMM) found on both *S. aureus* and *Staphylococcus epidermidis* [46]. In particular, fibrinogen binding proteins ClfA and SDrG (*S. epidermidis* serine-aspartate repeat protein G) are recognized by the IVIg. It is conceivable that a combination of well-characterized human monoclonal antibodies targeting *S. aureus* toxin(s) and surface antigens [28], [47], [48] may be a more useful immunotherapeutic for treating *S. aureus* infections. Furthermore, combinations of human anti-SEB monoclonal antibodies may be more effective than one, as previously demonstrated with monoclonal antibodies specific for *Clostridium botulinum* neurotoxin A [49] and tetanus toxin [50]. The use of SPR and different antibody combinations, or an ELISA with labeled monoclonal antibody and unlabeled competitor, could provide important insight into antibody combinations more useful in neutralizing SEB *in vivo* versus a single antibody. However, our studies showed that native Mabs 9 and 10 individually were more effective inhibitors of SEB than affinity-purified, polyclonal anti-SEB from human sera. A recent study by Tilahun et al. characterizes chimeric (human-mouse) monoclonal antibodies against SEB that are, like our findings with human monoclonal antibodies, not cross-reactive with SEA or TSST-1 [51]. Two of these chimeras recognize distinct epitopes, neutralize toxin *in vitro* and *in vivo*, and have a neutralizing synergy when combined against SEB *in vitro*.

In addition to *in vitro* characterization of the monoclonal antibodies in our study, biological assays involving human cell culture were used to predict the physiological (i.e. toxin neutralizing) effects of each antibody. The Fc domains added to Fabs 1 and 3 had minimal effects upon IC_50_s in cell culture, whereas native full-length Mabs 9 and 10 resulted in much higher inhibition versus their Fab counterparts. The increased efficacy of native Mabs 9 and 10 appeared dependent upon Fc receptors on mononuclear cells. Comparisons of any native full-length Mab with parental Fab revealed no significant differences in SEB-binding kinetics by SPR, and thus minimal disturbance of paratopes. Uniquely, antibodies 9 and 10 were the only two that significantly inhibited SEB binding to MHC II. Although it was plausible that Fc addition to Fabs 9 and 10 increased steric hindrance of toxin-TCR interactions, as reflected in the IC_50_ values, comparison of Fab with its native-length Mab revealed no differences in blocking SEB binding to MHC II. Fab 10 or its native Mab derivative were also the best performers in the mouse assay, thus agreeing with the predicted IC_50_s from cell culture. Additionally, mouse and non-human primate results from others describe antibody protection from SEB-induced toxic shock up to 4 h after toxin exposure [25], [26]. Such findings *in vivo* are congruent with our study using human cell culture and application of antibody after SEB. In fact, for one Fab (#1) there was noticeable protection (75%) 12 h post-SEB that was better than affinity-purified polyclonal antibody. Collectively, previous *in vivo* studies and the current cell-based efforts suggest that human monoclonal antibodies against SEB can act as potent neutralizers of toxin post exposure.

In addition to SEB, target specificity of each antibody was further examined with other staphylococcal and streptococcal superantigens by ELISA. Particularly noticeable was that many of the SEB-selected Fabs cross-reacted with SEC1 and/or SEC2. Similar results were previously demonstrated with rabbit antisera [9], [52] and mouse monoclonal antibodies [7], [53]. SEB shares the highest sequence identity with SECs (68%), versus SEA (33%) or TSST-1 (26%) [54], [55]. Vaccination of mice with either SEB or SEC1 protects against heterologous toxin, clearly suggesting common, neutralizing epitopes recognized by antibodies [10], [21]. Additionally, there was also significant cross-reactivity of Fabs 6, 9, and 10 with SpeC from *S. pyogenes*. However, this toxin shares less sequence homology (21%) with SEB compared to the non-reactive SpeA (52%). Perhaps the SpeC epitopes recognized by Fabs 6, 9, and 10 are more conformationally similar to SEB versus those on SpeA. Although ELISA cross-reactivity of the SEB affinity-purified antibodies from human sera with SEC1 and SpeC was equivalent, the IC_50_ was 10-fold higher for SEC1 versus SpeC. Our results revealed no cross-reactivity of any human monoclonal antibody or polyclonal SEB-specific IgG with SEA, SpeA, or TSST-1. Furthermore, each monoclonal antibody was also tested by Western blots using crude cell lysates and culture fluid of a SEB-producing strain of *S. aureus* as target antigen. There was high specificity of these antibodies (i.e. only one immunoreactive band evident), with exceptions being Fabs 4 and 5 that were not reactive and thus likely targeting conformationally-sensitive epitopes.

Immunoreactivity of at least one antibody was assay dependent as evidenced by Fab 4 and recognition of STEBVax, but not wild-type SEB, in an ELISA. This same antibody did not recognize SEB by Western blot. The difference between SEB and STEBVax consists of three residue changes which affect binding to MHC II. It is possible that adsorption of wild-type SEB onto an ELISA well distorted the epitope recognized by Fab 4, thus effectively preventing antibody-antigen interactions. However, results from IC_50_ and therapeutic experiments suggested that Fab 4 effectively interacted with SEB in solution. Overall, such data suggest binding differences among the tested antibodies and recognition of very subtle differences in amino acid sequences and/or conformations of SEB. Furthermore, the strategy of using a recombinantly-attenuated molecule of SEB (STEBVax) is potentially useful for other toxins in which there are concerns (biodefense or otherwise) linked to shipping and biocontainment.

Finally, polyclonal antibodies from pooled human sera are currently used to treat various diseases such as Kawasaki's syndrome, *Clostridium difficile* colitis, respiratory syncytial virus, cytomegalovirus, chronic dysimmune neuropathies, chronic inflammatory demyelinating polyradiculo-neuropathy, and polyarteritis nodosa [56]–[62]. These IVIg products naturally vary in batch-to-batch potency, represent a potential risk for transmitting infectious agents, and the mechanisms of action are generally not well characterized [31]. Recombinantly-engineered human monoclonal antibodies targeting specific virulence factors, as presented in this current study, circumvent many of these concerns.

## Materials and Methods

### Human Monoclonal Antibodies

SEB-specific antibodies were isolated from a phage-displayed, recombinant antibody library (MorphoSys AG, Martinsried, Germany) as described previously [39]. Briefly, each of the human variable heavy (V_H_) and light (V_L_) chain subfamilies frequently used during an immune response were represented by one consensus framework, thus resulting in seven master genes each for the V_H_ and V_L_ chains. Hypervariable genetic cassettes encoding the complementarity-determining regions were introduced into the framework sequences to create >1.2×10^10^ clones. Each cDNA encoded a bivalent Fab (110 kDa) containing a 5 kDa homodimerization domain (dHLX), fused to 6xHis and Myc tags at the C-terminus of the heavy chain to facilitate subsequent purification and detection. The panning antigen (STEBVax), previously developed as a vaccine [20]–[22], was a recombinant SEB harboring three site-specific mutations in residues comprising the MHC II binding surface. For panning, STEBVax was biotinylated and bound to streptavidin covalently attached to paramagnetic beads. After enrichment of SEB-specific phage, the antibody genes were subcloned as a pool into an *E. coli* expression vector [39]. Proteins from the transformants were screened for binding to SEB by ELISA, as described below. The concentration of each antibody specific for SEB was assessed by absorbance at 280 nm (A_280_) using an extinction coefficient of 0.7 for 1 mg/ml, and aliquots subsequently stored at −80°C. Endotoxin levels for each antibody were <1.5 units/ml as determined by a *Limulus* amebocyte lysate assay (BioWhittaker, Walkersville, MD). Select Fabs were converted into native full-length (FL) IgG (Mab) expressed in HEK cells, using the above technology [39], and then purified for additional study. Purity of the SEB-specific antibodies was confirmed by SDS-PAGE and Coomassie Blue staining.

### Polyclonal Human Anti-SEB Immunoglobulins

Polyclonal IgG, specific for SEB, was prepared from pooled human sera [29], [63]. Purified, wild-type SEB (Toxin Technology, Sarasota, FL) was coupled to cyanogen bromide-activated Sepharose 4B (Sigma Chemical Co., St. Louis, MO) and used as an affinity matrix. Semi-purified human IgG from sera (FFF Enterprises, Temecula, CA) was diluted to 1 mg/ml with phosphate buffered saline, pH 7.4 (PBS) and then passed over the SEB-containing column. After addition of semi-purified IgG, the column was washed with PBS until the A_280_ readings returned to baseline. SEB-specific IgG was eluted from the column with 0.1 M glycine buffer (pH 2.5) and dialyzed extensively against PBS. Protein concentrations were determined by a bicinchoninic acid assay (Pierce, Rockford, IL) with purified human IgG as a standard. Purity of the SEB-specific IgG was confirmed by SDS-PAGE and Coomassie Blue staining.

### Antibody Cross-Reactivity

Cross-reactivity of each Fab was tested by ELISA with various wild-type bacterial superantigens. Purified SEA, SEB, SEC1, SEC2, TSST-1, streptococcal pyrogenic exotoxin A (SpeA), and SpeC (Toxin Technology, Sarasota, FL) were each applied (5 µg/ml PBS) in triplicate to Costar 96-well flat bottom plates (Corning Inc., Corning, NY) for 2 h at 37°C. PBS containing 0.1% bovine serum albumin (BSA) was used as a negative control to establish baseline data. Plates were blocked (16 h, 4°C) with 0.2% casein in PBS and washed with PBS containing 0.05% Tween-20 using an ELx405 Select washer (BioTek Instruments, Winooski, VT). ELISA plate wells were incubated (90 min, 37°C) with either Fab (2 µg/ml) or the SEB affinity-purified IgG from human sera (20 µg/ml), diluted in PBS containing 0.02% casein. Plates were washed and incubated with goat anti-human F(ab)'2 – horseradish peroxidase conjugate (90 min, 37°C). Tetramethyl-benzidine substrate (Pierce) was added to each well and absorbance (650 nm) recorded at 40 min. Wells were scored positive if the mean absorbance +/− standard deviation of triplicates was greater than twice the baseline. Results are representative of three separate experiments.

Western blots were done to test binding specificity of each Fab using *S. aureus* lysate and culture supernatant as complex antigen mixtures. Previously characterized *S. aureus* (strain 14458) was grown overnight at 37°C in brain heart infusion broth [64]. Following centrifugation, protein from lysed cells (B-PER; Pierce) and culture supernatant were precipitated overnight by cold acetone (70%). The precipitate was centrifuged, protein pellet gently washed with Tris-buffered saline (TBS), and finally dissolved in TBS. Protein concentrations were determined by the bicinchoninic acid assay (Pierce). Proteins were separated by 10–20% gradient SDS-PAGE and transferred onto a nitrocellulose membrane (Invitrogen, Carlsbad, CA) that was then blocked overnight in TBS – 0.1% Tween 20 (TBST) containing 3% BSA. Each Fab (1 µg/ml in TBST) was then applied to the membrane for 1 h at room temperature and the latter subsequently washed (4×15 min) with TBST. There was a subsequent 1∶2000 dilution of goat anti-human F(ab)'2– horseradish peroxidase conjugate added to the membrane for 1 h at room temperature. Following TBST washes, immunoreactive bands were detected by electrochemiluminescence (Pierce).

### Kinetic Analysis of Toxin-Antibody Interactions

Interactions of antibodies with SEB were assessed by surface-plasmon resonance (SPR) using a Biacore 3000 instrument (Biacore Inc., Piscataway, NJ). Mouse anti-histidine tag (AbD Serotec, Dusseldorf, Germany) or goat anti-human Fc (GE Healthcare, Piscataway, NJ) antibodies were immobilized in 10 mM acetate buffer (pH 5.0) at high densities (8000 resonance units or RUs) on a CM5 sensor chip. The SEB antibodies were captured using increasing concentrations to reach an Rmax of analyte optimal for kinetic analysis (50–250 RUs). To measure association rates, dilutions of SEB (11–2700 nM) in 0.1 M HEPES buffer containing 1.5 M NaCl, 30 mM EDTA, and 0.5% P40 surfactant (pH 7.4, 25°C) were used at a flow rate of 50 µl/min (3 min total time). Running buffer (as above) was then injected (50 µl/min for 5 min) to assess the dissociation phase. Surfaces were regenerated using 10 mM glycine buffer (pH 1.8) or a 3M MgCl_2_ solution injected at 30 µl/min for 30 sec. The resulting sensorgrams were analyzed with BIAevaluation software to determine the association (ka) and dissociation (kd) rate constants, using a Langmuir 1∶1 binding model. The dissociation constant (KD) was calculated as kd/ka. Results are representative of two separate experiments.

### Antibody Inhibition of Toxin-induced Stimulation of T-cells: IC_50_ and Therapeutic Potential *in vitro*

Peripheral blood mononuclear cells (PBMCs) were isolated by Ficoll-hypaque density separation (460×g for 30 min at 4°C) from healthy human donors. The PBMC layer was removed, washed three times in RPMI medium, and cryopreserved in 10% dimethylsulfoxide plus 90% fetal calf serum (FCS) under liquid nitrogen. For T-cell assays, cryopreserved PBMCs were rapidly thawed (37°C water bath), gently washed twice in RPMI (37°C) containing 10% FCS, and dispersed into 96-well plates (10^5^cells/well). To establish the 50% inhibition concentration (IC_50_) of each antibody, cells were incubated (16 h, 37°C) in duplicate wells with 0.25–1500 nM antibody and concentrations of each toxin producing 50% maximum T-cell stimulation: SEB (0.35 nM), SEC1 (1.3 nM), or SpeC (467 nM). Negative and positive antibody controls respectively consisted of an SEA-specific Fab (not cross-reactive with SEB) and affinity-purified anti-SEB IgG from human sera. Media were collected from the cultures and released IFNγ used as a marker of T-cell activation. To test antibody blocking of SEB-induced stimulation of T cells after toxin exposure (i.e. therapeutic potential), each monoclonal antibody (1000 nM) was added to PBMCs at 1, 3, 5, 7, 9, or 12 h after SEB (0.35 nM). Duplicate wells were pooled and tested for IFNγ.

To measure cytokine levels, anti-human IFNγ capture antibody (Thermo Fisher, Waltham, MA), rabbit anti-SEA antibody (negative control; Toxin Technology), and Alexa647-labeled streptavidin (positive control; Invitrogen) were arrayed onto PATH Protein Microarray slides (Gentel Bioscience, Madison, WI) using an inkjet microarray spotter (ArrayJet, Roslin, Scotland, UK). The microarrayed slides were blocked (2 h, 22°C) in 50 mM HEPES buffer (pH 7.4) containing 200 mM NaCl, 0.08% Triton X-100 and 50% glycerol. Culture supernatants diluted 1∶3 in sample buffer (1% BSA +0.1% Tween-20 in PBS) were incubated on the surface of each slide (1.5 h, 22°C). Media from PBMC cultures stimulated for 24 h with 2 µg/ml phytohemagglutinin (Invitrogen) were calibrated against an IFNγ standard (National Cancer Institute, Frederick, MD) using a cytometric bead assay (BD Biosciences, San Jose, CA). Following washes in 1% BSA +2% Tween-20 in PBS, slides were incubated (1 h, 22°C) with 1 µg/ml of biotinylated anti-human IFNγ (Invitrogen), washed and developed (1 h, 22°C) with Alexa647-labeled streptavidin (1 µg/ml). The microarrays were washed to remove non-bound streptavidin, dried, and fluorescent binding events measured by a GenePix 4000B. The IC_50_ was calculated relative to SEB-treated PBMCs without antibody. The therapeutic potential of each antibody was plotted as percent inhibition over time. Results are representative of two experiments with separate donors.

Mouse L-cells were used to examine the role of Fc receptors in toxin neutralization by native full-length Mabs. These cells, which do not express endogenous Fc receptors, were transfected with the genes for human MHC II proteins (HLA-DR1α/β) and used to present SEB to T-cells as described elsewhere [65], and above.

### Antibody Inhibition of SEB Binding to MHC II

The HLA-DR1 homozygous B lymphoblast line, LG2, was used to detect antibodies that inhibited SEB - MHC II (HLA-DR, -DQ, -DP) interactions. SEB (1 µM) was incubated (30 min, 37°C) with antibodies (0.5–5 µM) in Dulbecco's Modified Eagle's Medium (DMEM) before addition to cells in 96-well plates. The cells (5×10^5^/well) were cultured (20 min, 37°C, 5% CO_2_) in DMEM containing 10% FCS. Positive and negative controls included SEB incubated with either affinity-purified anti-SEB IgG from human sera or anti-SEA Fab (5 µM), respectively. Following a DMEM wash (4°C), cells were incubated (30 min, 4°C) with a 1∶50 dilution of rabbit anti-SEB (Toxin Technologies). Additional washes in DMEM were followed by a 1∶25 dilution of goat anti-rabbit antibody conjugated to fluorescein isothiocyanate (Pharminogen, San Jose, CA). After a final wash, cells were fixed in 1% paraformaldehyde and SEB binding was determined by flow cytometry (FACSCalibur, Becton Dickinson, Mountain View, CA). Percent inhibition was calculated by comparison of antibody-treated cells incubated with SEB, but no monoclonal antibody, and all other reagents (0% inhibition). Results are indicative of three separate experiments.

### Antibody Inhibition of Toxic Shock

Finally, antibodies were tested *in vivo* for SEB neutralization in a toxic shock model involving synergy of bacterial superantigens with lipopolysaccharide [23]–[25]. Each BALB/c mouse (20 g females purchased from the National Cancer Institute, Frederick, MD) was injected intraperitoneally with SEB (2.5 µg) previously incubated for 30 min at 22°C with antibody (10 µg). Mice were housed in a pathogen-free environment with food and water supplied *ad libitum*. Survival was recorded 72 h after injection and each test group consisted of six animals, unless stated otherwise.

### Ethics Statement

Blood was collected from healthy human volunteers (anonymous) at the U.S. National Institutes of Health for the preparation of PBMCs, following written consent in accordance with the Institutional Review Board guidelines. This is a general protocol for the collection of normal blood products for research purposes only.
